# Specific absorption rate of randomly oriented magnetic nanoparticles in a static magnetic field

**DOI:** 10.3762/bjnano.14.39

**Published:** 2023-04-14

**Authors:** Ruslan Alekseevich Rytov, Nikolai Aleksandrovich Usov

**Affiliations:** 1 National University of Science and Technology «MISiS», Moscow, Russia; 2 Pushkov Institute of Terrestrial Magnetism, Ionosphere and Radio Wave Propagation, Russian Academy of Sciences, IZMIRAN, Troitsk, Moscow, Russia

**Keywords:** dynamic hysteresis loop, magnetic hyperthermia, magnetic nanoparticles, magnetic particle imaging, specific absorption rate, static magnetic field

## Abstract

Numerical simulations using the stochastic Landau–Lifshitz equation are performed to study magnetization dynamics of dilute assemblies of iron oxide nanoparticles exposed to an alternating (ac) magnetic field with an amplitude *H*_ac_ = 200 Oe and a frequency *f* = 300 kHz and a static (dc) magnetic field in the range *H*_dc_ = 0–800 Oe. The specific absorption rate (SAR) of the assemblies is calculated depending on the angle between the directions of the ac and dc magnetic fields. For the case of an inhomogeneous dc magnetic field created by two opposite magnetic fluxes, the spatial distribution of the SAR in the vicinity of the field-free point is obtained for assemblies with different nanoparticle size distributions. The results obtained seem to be helpful for the development of a promising joint application of magnetic nanoparticle imaging and magnetic hyperthermia.

## Introduction

Magnetic nanoparticles, mainly iron oxides, are promising materials for the diagnosis and therapy of oncological diseases [1–3]. Important fields of application of magnetic nanoparticles in biomedicine are magnetic particle imaging (MPI) [4–6] and magnetic hyperthermia (MH) [1–2,6–7]. Magnetic hyperthermia uses the ability of magnetic nanoparticles to generate heat under the influence of an external alternating (ac) magnetic field of moderate frequency, *f* = 200–400 kHz, and amplitude, *H*_ac_ = 100–200 Oe [1,7–8]. In magnetic hyperthermia, magnetic nanoparticles are introduced into the tumor and heated by absorbing the energy of the ac magnetic field. The intensity of heat release is characterized by the specific absorption rate (SAR) of an assembly. Maintaining a temperature in the tumor in the range of 41–43 °C over several medical treatments leads to the tumor destruction, as well as to the activation of the body’s immune response to cancer cells [8]. However, the introduction of MH into clinical practice is hindered by a number of difficulties. Unfortunately, it is not easy to control the distribution of magnetic nanoparticles in a biological environment, nor is it to monitor the temperature distribution in the heated area [1–2,8].

It is assumed [6,8–12] that some of these problems can be overcome by combining MPI and MH techniques. The MPI-MH combination will make it possible to monitor the distribution of nanoparticles in living tissues during MH. In addition, by controlling the spatial distribution of a non-uniform dc magnetic field, it is possible to suppress the SAR in the entire range of action of the ac magnetic field in a biological environment, except for a certain area near the field-free point (FFP). This will allow one to localize the heat release in the tumor area with millimeter accuracy. The search for optimal assemblies for joint MH-MPI therapy is an urgent task in this research area.

Experimental measurements of dynamic hysteresis loops of assemblies under the influence of ac and dc magnetic fields were carried out in [13–14]. It was shown [13] that an application of dc magnetic fields with *H*_dc_ ≥ 400 Oe is sufficient to completely suppress the SAR of an assembly of FeCo nanoparticles. It was also found [14] that for magnetic nanoparticles in a liquid, an increase of the dc magnetic field leads to a decrease in the area of the hysteresis loop for both parallel and perpendicular configurations of external magnetic fields.

Analytical and numerical calculations of the dynamics of the nanoparticle magnetization in an ac magnetic field in the presence of a dc field were carried out in [15–21]. In particular, the behavior of assemblies of nanoparticles distributed in a viscous liquid was considered in [18–21]. However, nanoparticles in a liquid show a more complex behavior because of the presence of both magnetic and mechanical degrees of freedom of the particles [17,22–23]. Meanwhile, in a biological environment, the rotation of magnetic nanoparticles as a whole under the action of ac magnetic field is strongly hindered [24], so that the spatial orientation of nanoparticles in biological media can be considered fixed and random. In this case, the dynamics of the assembly magnetization depends on the parameters of the external magnetic field, as well as on the magnetic and geometric parameters of an assembly [20,22,25–26].

To develop the joint MPI-MH technique, it is necessary to study in detail the dependence of the SAR of a randomly oriented assembly on the magnitude and direction of the external dc field with respect to that of the uniform ac field. In this work, using the numerical solution of the stochastic Landau–Lifshitz equation [17,20,22–23,26], the dynamics of non-interacting magnetic nanoparticles of iron oxide is considered in a wide range of particle diameters, *D* = 18–50 nm. The SAR of the assembly is calculated depending on the amplitude and direction of the dc magnetic field in the range *H*_dc_ = 0–800 Oe. The spatial distribution of the assembly SAR in the vicinity of the FFP has been obtained considering an inhomogeneous dc magnetic field created by two opposite magnetic fluxes, for the case of assemblies with different nanoparticle size distributions.

## Results and Discussion

### Numerical simulation

Let us consider a dilute assembly of magnetic nanoparticles with uniaxial magnetic anisotropy randomly oriented in a solid matrix. The saturation magnetization of particles and the magnetic anisotropy constant are taken to be *M*_s_ = 350 emu/cm^3^ and *K*_1_ = 10^5^ erg/cm^3^, respectively [27]. The range of nanoparticle diameters studied is *D* = 18–50 nm, the temperature of the system is *T* = 300 K. Let the assembly of nanoparticles be in an ac magnetic field with an amplitude *H*_ac_ = 200 Oe and a frequency *f* = 300 kHz. These ac field parameters are typical for the use in magnetic hyperthermia [1,7–8]. In this work, we are interested in the dependence of the assembly SAR on the intensity of the dc external magnetic field additionally applied to the assembly. It is assumed that the dc magnetic field varies in the range *H*_dc_ = 0–800 Oe and is applied at different angles to the ac magnetic field direction. The behavior of a randomly oriented assembly under the combined action of ac and dc magnetic fields is studied using a numerical simulation based on the solution of the stochastic Landau–Lifshitz equation [17,20,22–23,26]. The SAR of a randomly oriented assembly is calculated in terms of the area of the dynamic hysteresis loop according to the well-known formula [28–29]




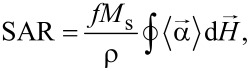




where ρ is the density of the magnetic material and 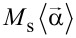 is the average magnetization of the assembly of nanoparticles. To obtain statistically reliable results, dynamic hysteresis loops are averaged over 30 independent realizations of the randomly oriented assembly containing 60 non-interacting nanoparticles of a fixed diameter.

### *H*_dc_ is parallel to *H*_ac_

**Figure 1:**
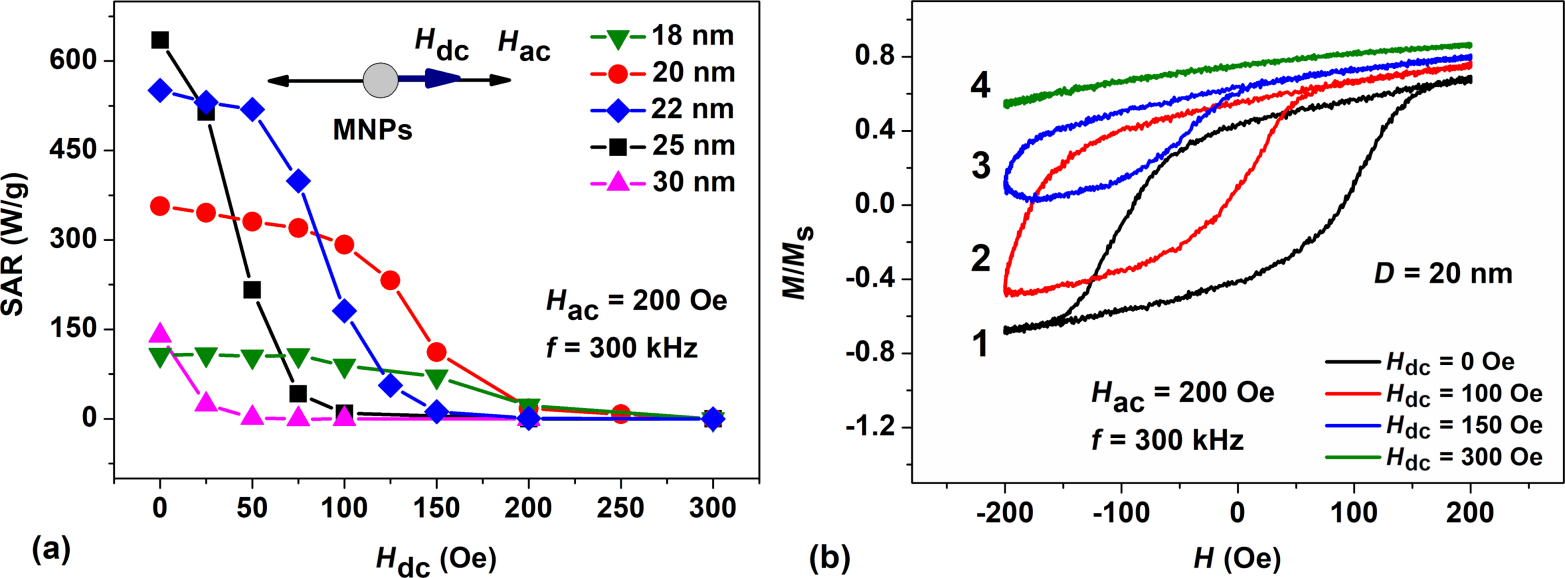
(a) Dependence of the SAR of randomly oriented assemblies of nanoparticles of various diameters distributed in a solid matrix on a dc magnetic field *H*_dc_ applied parallel to the ac field *H*_ac_. (b) Dynamic hysteresis loops for an assembly of nanoparticles with *D* = 20 nm at different *H*_dc_ values: (1) *H*_dc_ = 0, (2) *H*_dc_ = 100 Oe, (3) *H*_dc_ = 150 Oe, and (4) *H*_dc_ = 300 Oe.

Let us first consider the case when the external dc magnetic field in the range *H*_dc_ = 0–300 Oe is applied parallel to the direction of the ac magnetic field with amplitude *H*_ac_ = 200 Oe. The results of the SAR calculation for a dilute, randomly oriented assembly depending on the value of the dc magnetic field are shown in Figure 1.

As Figure 1a shows, in the absence of a dc magnetic field, *H*_dc_ = 0, there is an interval of optimal particle diameters, *D* = 18–30 nm, where the SAR of the assembly exceeds 100 W/g at the given frequency and amplitude of the ac magnetic field. Outside this optimal window, SAR values are close to zero. Hence, SAR values for particles with *D* < 18 nm and *D* > 30 nm are not shown in Figure 1a. Applying a dc magnetic field parallel to the ac field direction results in a significant SAR decrease. According to Figure 1a, to completely suppress the SAR of an assembly in a given field configuration, it is sufficient to fulfil the inequality *H*_dc_ ≥ *H*_ac_ = 200 Oe, regardless of the particle diameter. However, the behavior of the function SAR(*H*_dc_) significantly depends on the nanoparticle diameter. For example, for particles with a diameter *D* < 22 nm, a dc field of *H*_dc_ < 100 Oe has a relatively weak effect on the SAR, but the SAR decreases rapidly at *H*_dc_ > 100 Oe. In contrast, for particles with *D* = 25 nm, which show the maximum SAR at *H*_dc_ = 0, even a relatively weak dc field, *H*_dc_ = 50 Oe, causes a significant drop in the SAR of the assembly.

Figure 1b shows the change in the shape of the dynamic hysteresis loop with increasing *H*_dc_ for a particular case of a randomly oriented assembly of nanoparticles with *D* = 20 nm. It can be seen that the hysteresis loop is strongly deformed as a function of *H*_dc_. For positive values of the ac magnetic field, when the fields *H*_ac_(*t*) and *H*_dc_ add up, the hysteresis loop of the assembly actually collapses. However, for negative values of *H*_ac_(*t*), when the ac and dc fields compensate each other, the loop area decreases significantly. Furthermore, with an increase in the amplitude of the dc field, the average magnetization of the assembly gradually increases, which leads in Figure 1b to an upward shift of the dynamic hysteresis loop with increasing of *H*_dc_. Finally, at *H*_dc_ ≥ 300 Oe the hysteresis loop collapses completely. Evidently, the absorption of the ac magnetic field energy in a sufficiently strong dc field is absent, since magnetization reversal of nanoparticles is impossible.

### *H*_dc_ is perpendicular to *H*_ac_

**Figure 2:**
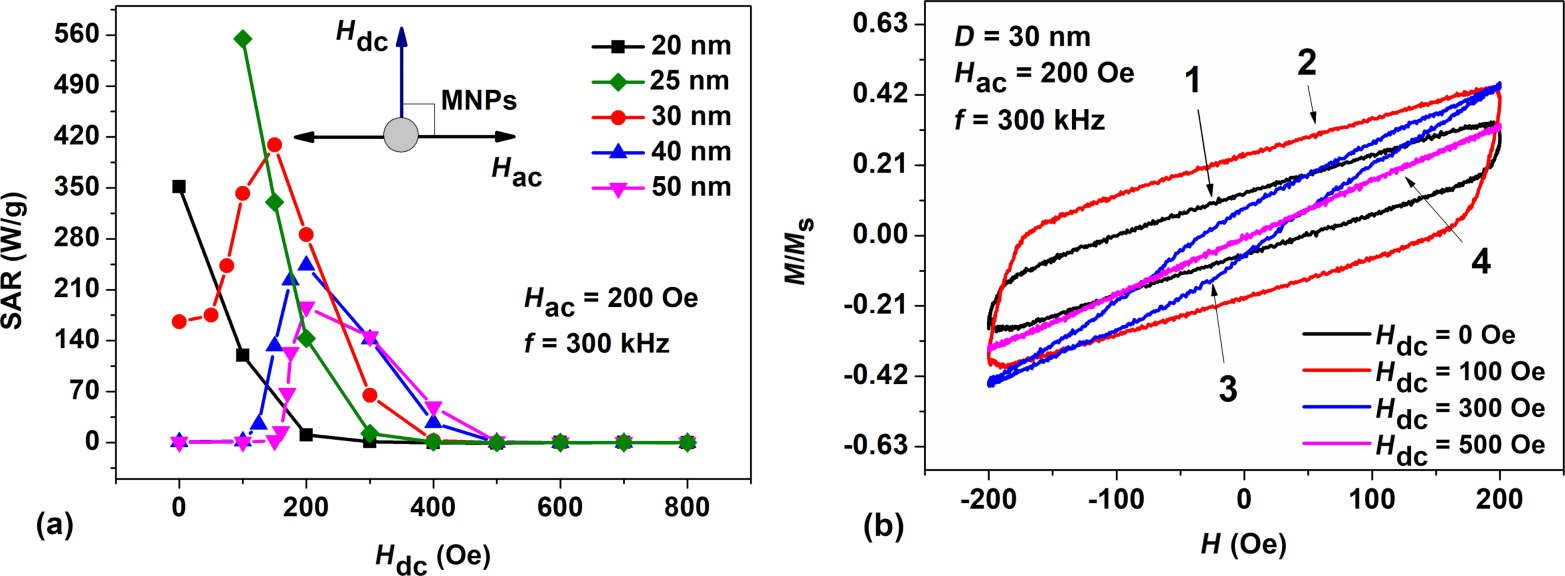
(a) Dependence of the SAR on the dc magnetic field applied perpendicular to the ac magnetic field for randomly oriented assemblies of nanoparticles of various diameters. (b) Dynamic hysteresis loops for an assembly of nanoparticles with *D* = 30 nm as a function of *H*_dc_: (1) *H*_dc_ = 0, (2) *H*_dc_ = 100 Oe, (3) *H*_dc_ = 300 Oe, and (4) *H*_dc_ = 500 Oe.

Let us now consider the case of perpendicular orientation of the external dc and the ac magnetic fields. The dc magnetic field changes in this case in the range *H*_dc_ = 0–800 Oe. The results of SAR calculation as a function of *H*_dc_ for randomly oriented assemblies of nanoparticles with diameters *D* = 20–50 nm, where SAR values are non-zero, are shown in Figure 2.

As Figure 2a shows, the SAR of assemblies of nanoparticles with *D* = 20 and 25 nm rapidly decreases with increasing *H*_dc_. In fact, for particles of these diameters the SAR of the assembly is completely suppressed at *H*_dc_ ≥ 300 Oe. It is interesting to note, however, that in contrast to the parallel field configuration, in the given case an interval of values *H*_dc_ = 100–300 Oe appears, where the SAR of the assembly increases sharply for nanoparticles with *D* = 30–50 nm. For example, as Figure 2a shows, for the assembly with *D* = 30 nm the SAR rapidly increases at *H*_dc_ > 50 Oe, reaches a maximum of 410 W/g at *H*_dc_ = 150 Oe, and then gradually decreases with a further increase in *H*_dc_. Complete suppression of the SAR for assemblies of particles with *D* = 30–50 nm occurs in this case only at *H*_dc_ ≥ 500 Oe.

Figure 2b shows the shape of dynamic hysteresis loops for nanoparticles with *D* = 30 nm at different *H*_dc_ values. In the absence of a dc field, *H*_dc_ = 0, the loop area is finite, and according to Figure 2a, the SAR of the assembly is 160 W/g. However, as the dc field increases to *H*_dc_ = 100 Oe, the dynamic hysteresis loop of the assembly expands and the SAR of the assembly increases to 350 W/g. However, with a further increase in *H*_dc_, the area of the hysteresis loop gradually decreases, and, finally, the loop collapses completely at *H*_dc_ = 400 Oe. The functions SAR(*H*_dc_) for randomly oriented assemblies of nanoparticles with diameters *D* = 40 and 50 nm behave similarly.

**Figure 3:**
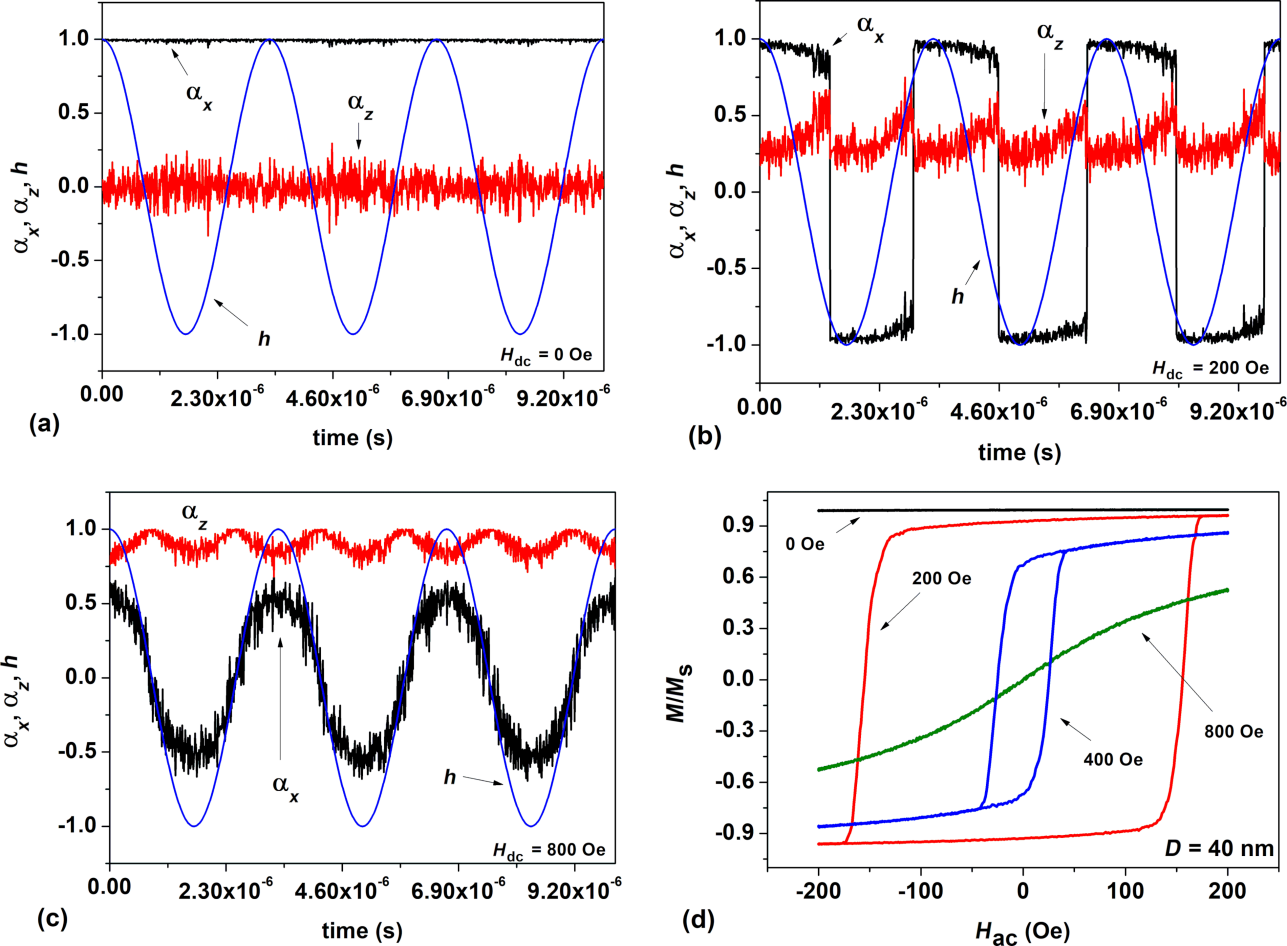
(a) Dynamics of α*_x_* and α*_z_* components of the unit magnetization vector of a nanoparticle with *D* = 30 nm at *H*_dc_ = 0; *h* marks the time dependence of the normalized ac magnetic field directed along the *x* axis. (b) The same in a dc field of *H*_dc_ = 200 Oe directed along the *z* axis, perpendicular to the particle easy anisotropy axis. (c) The same for *H*_dc_ = 800 Oe. (d) Dynamic hysteresis loops of the nanoparticle for *H*_dc_ = 0, 200, 400, and 800 Oe, respectively.

To explain this effect, Figure 3 shows the magnetization dynamics of a nanoparticle with *D* = 30 nm and with the easy anisotropy axis parallel to the ac field direction. The latter is parallel to the *x* axis, whereas the dc magnetic field is assumed to be directed along the *z* axis (see inset in Figure 2a). Figure 3a–c shows the dynamics of the α*_x_* and α*_z_* components of the particle unit magnetization vector, as well as the time dependence of the normalized ac magnetic field, denoted by the symbol *h*. It is assumed that at the initial moment of time, the unit magnetization vector of the particle is given by α*_x_* = 1. Irregular perturbations of the components of the unit magnetization vector occur due to thermal fluctuations of the particle magnetic moment at *T* = 300 K.

As Figure 3a shows, in the absence of a dc field, *H*_dc_ = 0, the unit magnetization vector of the particle fluctuates near the bottom of the potential well, α*_x_* ≈ 1, α*_z_* ≈ 0. However, according to Figure 3b, as the dc field increases to *H*_dc_ = 200 Oe, due to a change in particle energy barrier, the α*_x_* component begins to jump between particle potential wells alternately taking values of ±1. Besides, when the ac magnetic field is small, *h* ≈ 0, the α*_z_* component rapidly increases and reaches values of α*_z_* ≥ 0.5. As Figure 3d shows, at *H*_dc_ = 200 Oe, the dynamic hysteresis loop of the particle becomes almost rectangular. As a result, the assembly SAR has a maximum at a given value of *H*_dc_. With a further increase in *H*_dc_, the dc field begins to dominate over the ac field, so that at *H*_dc_ = 800 Oe the unit magnetization vector of the particle turns out to be mainly directed along the *z* axis, perpendicular to the ac magnetic field (see Figure 3c). As Figure 3d shows, this leads to a sharp drop in the particle hysteresis loop area.

**Figure 4:**
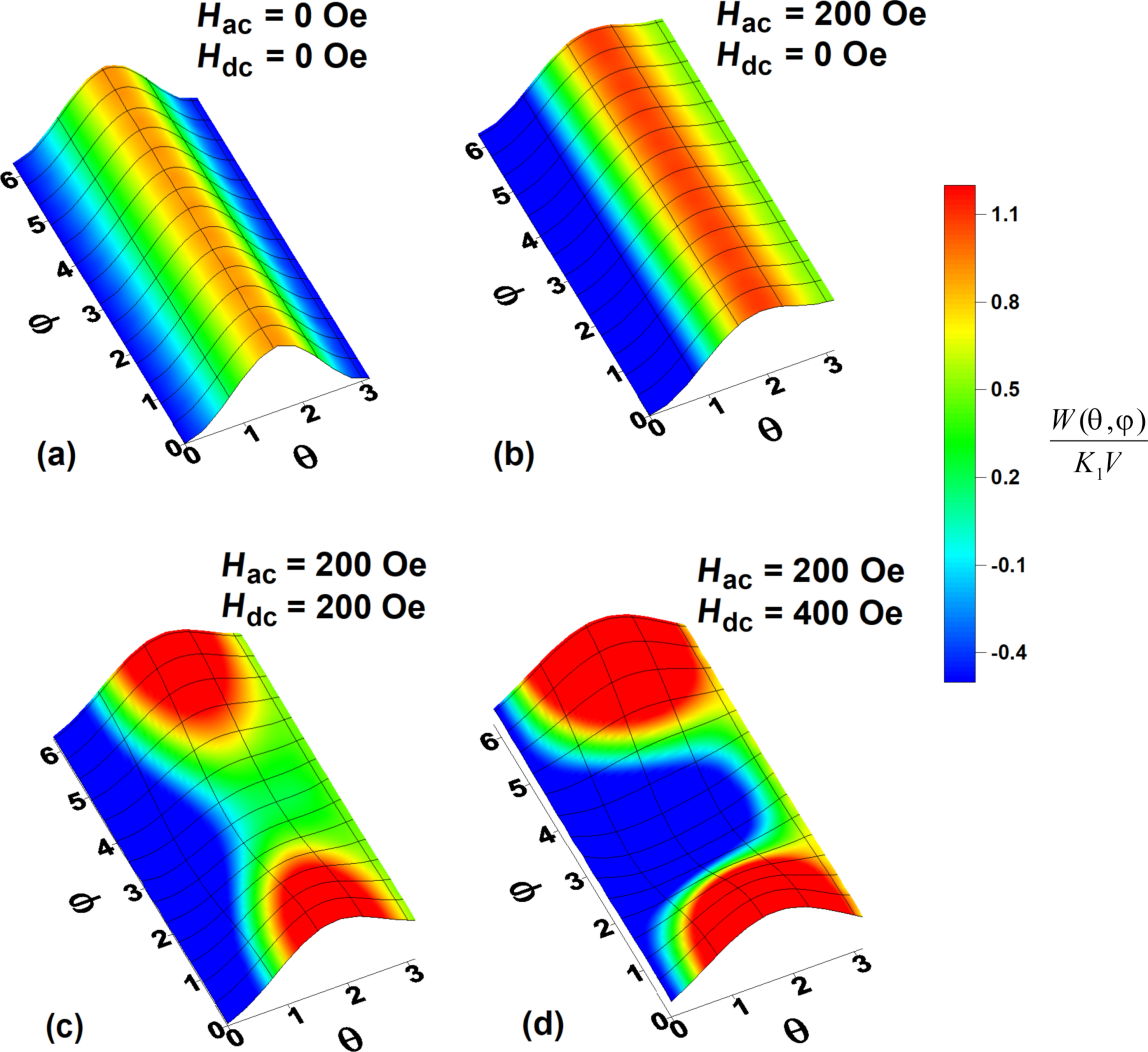
Evolution of the normalized energy density of a particle, *W*(θ,φ)/*K*_1_*V*, as a function of dc magnetic field applied perpendicular to the particle easy anisotropy axis: (a, b) *H*_dc_ = 0 Oe, (c) *H*_dc_ = 200 Oe, and (d) *H*_dc_ = 400 Oe.

In addition to Figure 3, Figure 4 shows the evolution of the surface of the normalized energy density of a nanoparticle, *W*(θ,φ)/*K*_1_*V*, as a function of the spherical angles θ and φ (in radians) in a dc magnetic field directed perpendicular to the particle easy anisotropy axis.

Figure 4a and Figure 4b show the total energy of the particle in the absence of a dc magnetic field, *H*_dc_ = 0, at times when the current ac magnetic field is zero or equal to its maximum value, *H*_ac_ = 200 Oe. Obviously, at *H*_dc_ = 0, the potential wells of the nanoparticle located at θ = 0 and θ = π are separated by a high potential barrier, which is independent of the angle φ. However, if the perpendicular dc magnetic field increases to *H*_dc_ = 200 Oe, a saddle trajectory appears on the barrier at φ = π (see Figure 4c). As a result, the height of the energy barrier between the potential wells decreases significantly. This leads to an increase in the probability of magnetization reversal of nanoparticles of large diameters, *D* = 30–50 nm. At *H*_dc_ = 200 Oe, this gives an almost rectangular hysteresis loop, shown in Figure 3d. However, in a sufficiently strong perpendicular magnetic field, *H*_dc_ = 400 Oe (Figure 4d), a deep potential well appears in which the magnetic moment of the particle is actually blocked, α*_x_* ≈ 0, α*_z_* ≈ 1, since the direction of the dc magnetic field becomes energetically beneficial.

### Angle-dependence of SAR

It is important to note that a saddle between potential wells also arises when a dc magnetic field is directed at some angle to the particle easy anisotropy axis. As a result, for particles of sufficiently large diameters, *D* = 30–50 nm, oriented at a certain angle to the direction of the dc magnetic field of sufficient magnitude, a significant decrease in the energy barriers occurs. It is these particles that make the main contribution to the increase in the assembly SAR with increasing *H*_dc_, as Figure 2a shows. For smaller diameter particles, *D* < 25 nm, a dc field only suppresses the SAR value, since the energy barriers for such particles are relatively small.

### Application to MPI

During the joint implementation of MPI and MH, the assembly of magnetic nanoparticles will be in a uniform ac magnetic field, as well as in a dc field with a complex spatial distribution. As shown above, a dc magnetic field is capable of both suppressing and increasing the SAR of an assembly, depending on the magnitude and relative orientation of the ac and dc magnetic fields, while the characteristic diameter of the magnetic nanoparticles is also important.

**Figure 5:**
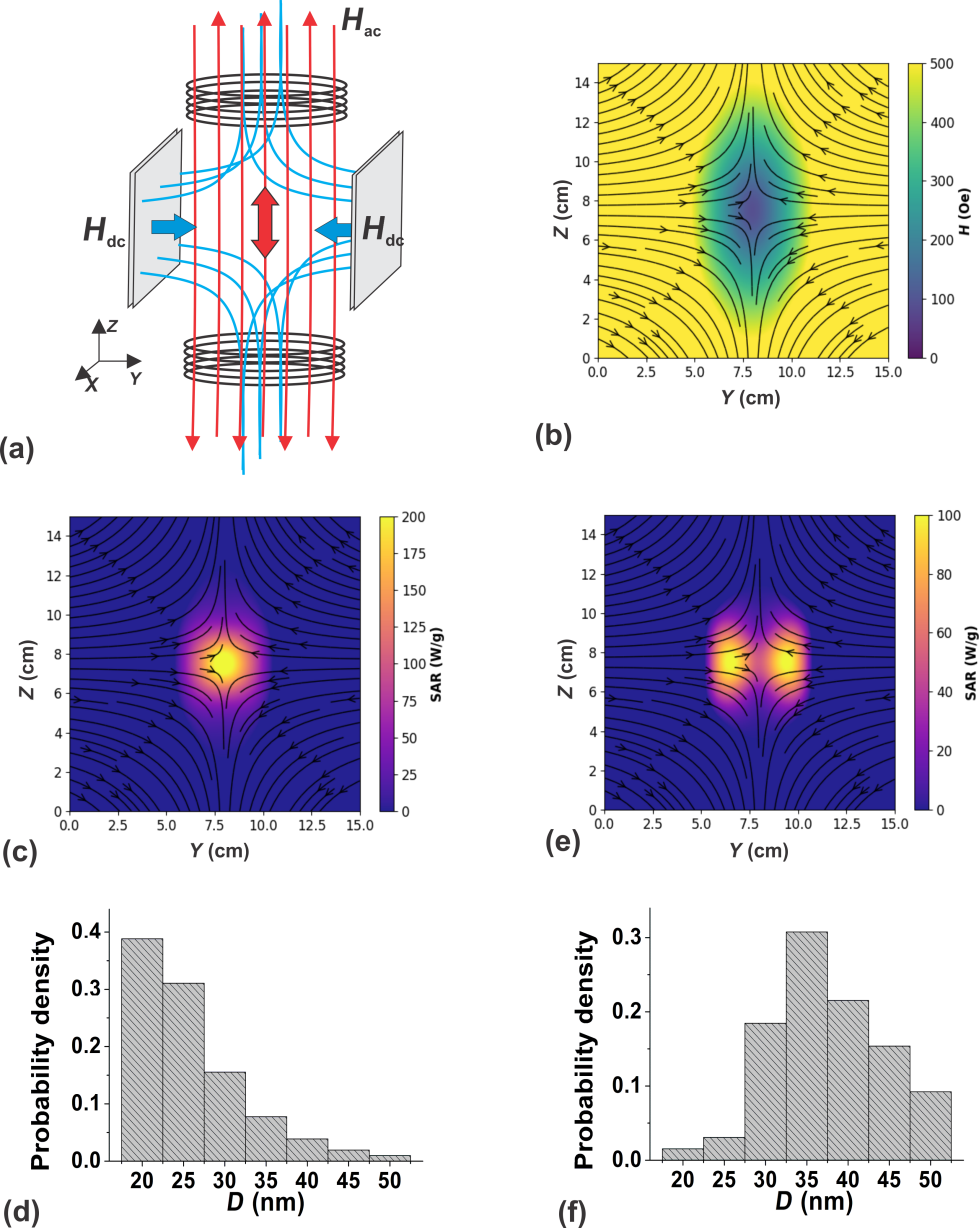
(a) Schematic representation of the crossed uniform ac and non-uniform dc magnetic field geometry with a FFP at the center of the system; (b) distribution of the amplitude and direction of the inhomogeneous dc field in the plane *yz*, near the center of the system shown in Figure 5a; (c) SAR distribution in the vicinity of the FFP in the *yz* plane for an assembly of fine nanoparticles (d); (e) the same for coarse nanoparticles (f).

In this section, as an example of a possible SAR spatial distribution, we consider the situation when a polydisperse assembly of nanoparticles is in an inhomogeneous dc magnetic field formed by two opposite magnetic fluxes. The assembly is excited by a uniform ac field created by Helmholtz coils. The SAR calculation is carried out in the vicinity of the corresponding FFP. The geometry of the system under consideration is schematically shown in Figure 5a.

Figure 5b shows the distribution of the magnitude and direction of a non-uniform dc magnetic field created by two opposite magnetic fluxes in the *yz* plane in the vicinity of the FFP. The maximum value of the dc field outside the region under consideration is *H*_dc_ ≈ 800 Oe. However, it rapidly decreases to zero as the FFP is approached. A uniform ac magnetic field with amplitude *H*_ac_ = 200 Oe and frequency *f* = 300 kHz is directed along the *z* axis. As Figure 5b shows, in the *yz* plane the dc field direction turns out to be nearly perpendicular to the ac magnetic field in almost the entire region shown. Only near the center of the system, where the dc magnetic field is small, does it form a varying angle with the ac magnetic field direction.

Let the entire area shown in Figure 5b be filled with an immobile nonmagnetic medium containing a polydisperse assembly of randomly oriented magnetic nanoparticles. Let us first consider an assembly predominantly consisting of nanoparticles with *D* < 25 nm. The results of the calculation of local SAR values for this assembly in the geometry of the dc magnetic field, shown in Figure 5b, are presented in Figure 5с. The particle size distribution for this assembly is given in Figure 5d. It is easy to see that in the given case only particles located in a small cylindrical region near the FFP with a radius of about 1 cm are capable of effectively absorbing the energy of the ac magnetic field. Furthermore, near the FFP the assembly SAR reaches 200 W/g.

However, if the assembly of nanoparticles contains a large fraction of nanoparticles with the particle size distribution shown in Figure 5f, the spatial SAR distribution near the FFP changes. Figure 5e shows that in this case, the SAR of the polydisperse assembly does not exceed 100 W/g and is nonzero in a toroid structure with a radius of about 2 cm centered at the FFP. It is important to note also that in this case, near the FFP itself, the SAR is minimal, since the presence of a sufficiently strong dc magnetic field is necessary to activate the fraction of big magnetic nanoparticles.

The above example shows that in order to implement the combined MPI-MH method, it is necessary to first calculate the local distribution of SAR in the biological environment, taking into account the geometry and magnitude of dc and ac magnetic fields, as well as the size distribution in an assembly of magnetic nanoparticles. In particular, in the considered example, it seems preferable to use an assembly of nanoparticles with a narrow size distribution in the range *D* = 20–25 nm.

## Conclusion

It is known [1–2,7] that optimized assemblies of magnetic nanoparticles are promising for the use in magnetic hyperthermia. However, it is necessary to strictly control the spatial distribution of magnetic nanoparticles in the tumor in order to exclude undesirable thermal effects on healthy tissues surrounding the tumor [30]. The combination of MH and MPI techniques seems promising, as it will allow one to localize the heat release in the tumor-affected organ more accurately and to minimize the thermal effect on healthy tissues, which may also contain a certain amount of magnetic nanoparticles. Obviously, to implement this approach, it is necessary to study the thermal capacity of the assembly distributed in a biological medium under simultaneous action of uniform ac and inhomogeneous dc field, depending on the field geometry, the magnetic parameters of the nanoparticles, and their characteristic sizes.

In this paper, numerical simulations of the stochastic Landau–Lifshitz equation are used to study the dynamics of magnetization in dilute, randomly oriented assemblies of iron oxide nanoparticles under the combined action of ac and dc magnetic fields. It is shown that for nanoparticles with a diameter *D* < 25 nm, the SAR of the assembly monotonically decreases with increasing *H*_dc_, regardless of the angle between the ac and dc fields. Complete suppression of the SAR in this case occurs at *H*_dc_ ≥ *H*_ac_. Therefore, iron oxide nanoparticles with diameters *D* = 20–25 nm seem preferable for use in combined MPI-MH therapy since, in this case, the maximum heat release is concentrated in a well-localized region near the FFP.

Also, it has been found that for nanoparticles of larger diameters, *D* ≥ 30 nm, the change in the assembly SAR with an increase in the dc magnetic field has a nonmonotonic character. Namely, in the range *H*_dc_ = 100–300 Oe, the SAR of randomly oriented assemblies can increase appreciably, since a dc magnetic field significantly lowers the energy barriers between potential wells for nanoparticles whose easy axes are oriented at finite angles to the dc field direction. As a result, for larger nanoparticles, *D* > 30 nm, a significant decrease in SAR can occur near the FFP, where the dc field is low, since the magnetization reversal of large particles in this region is prohibited.
